# News and Notes

**Published:** 1907-04

**Authors:** 


### NEWS AND NOTES.

The germs of bubonic plague, it is claimed, have been imported in Oriental rugs.

The osteopaths in Texas are making active steps to secure from the legislature a bill establishing a board of osteopathic examiners.

February the 22nd, the University of Michigan celebrated the fifty-seventh anniversary of the medical department of this institution.

Seven German universities admit women on the same terms as men, and at present they have a total register of 116 women in the medical departments. This is an increase of 56 over the preceding year.

The Alabama Legislature has passed a bill, which will also probably pass the Senate, appropriating $50,000 to the Medical College of Alabama.

It is reported that work will soon begin upon the proposed $100,000 building for the Medical Department of the University of Louisiana, at New Orleans.

Definite plans are being made for the establishment of a larger Presbyterian hospital in Atlanta. The proposed building alone is to cost not less than $250,000.

Johns Hopkins University has received from the estate of Charles L. Marburg the sum of $150,000, of which amount $100,000 goes to the hospital and $50,000 to the university.

Dr. Everett H. Martin, of Kansas City, Mo., was shot and killed on February 9th in his office, presumably by a patient, Miss Maud Slater, who appears then to have killed herself. Jealously is supposed to have prompted the deed.

---

The St. Lazare prison, of Paris, which was built in the eleventh century as a leper's hospital, and has been one of the most interesting landmarks, is to be torn down and its site covered with open squares and five modern buildings.

---

The Austrian government has attempted to popularize nicotine free tobacco in order to lessen the harm from its use. It was found, however, that the accustomed pleasure of smoking was lost unless a certain per cent. of the nicotine was retained.

---

The Walker County Society, at its annual meeting held at Jasper, elected officers for 1907 as follows: President, Dr. Geo. S. Gilder, Carbon Hill, Ala.; Vice-president, Dr. H. J. Sankey, Nauvoo, Ala.; Secretary, Dr. Titus Manasco, Carbon Hill, Ala.; Censor, Dr. Wm. M. Cunningham.

---

The largest drug store in the world is in Moscow and is 203 years old. They make up about 2,000 prescriptions a day, and have a staff of 700 people. The organization is perfect and they rarely make any mistakes.—State Board Journal, February, 1907.

---

The Douglas County Medical Society recently convened and elected the following officers for the ensuing year Dr. T. R. Whitley, Douglasville, Ga., President; Dr. W. K. Burnett, Winn, Ga., Vice-president; Dr. J. L. Housworth, Bill Arp, Ga., Secretary and Treasurer. In addition to this, they decided to invite the Fifth District Medical Society to meet with them in Douglasville in July and promise to give them a good entertainment.

